# Prescribing Pattern of Antidiabetic Drugs in Indore City Hospital

**DOI:** 10.4103/0250-474X.45404

**Published:** 2008

**Authors:** Sudha Vengurlekar, Prerna Shukla, P. Patidar, R. Bafna, S. Jain

**Affiliations:** Smriti College of Pharmaceutical Education, 4/1, Piplia Kumar Kakad Mayakhedi Road, Indore-452 001, India

**Keywords:** Diabetes, inpatient, outpatient, Prescription Auditing

## Abstract

Diabetes mellitus is a metabolic disorder characterized by hyperglycemia, glycosuria, and sometimes ketonemia. The present study was carried out to assess prescribing practice and general trend of diabetes among patients at the Bombay Hospital, Indore. Prescriptions and complete records of diabetic patients were monitored and data was filed as per WHO prescription proforma. The study revealed that prescription of metformin (27%) and glimepiride (22.60%) were found to be maximum among various available antidiabetic drugs. Category wise the maximum prescribed drugs are glimepride (22.60%, sulfonylurea category), metformin (27%, biguanide category) and pioglitazones (13.90%, glitazone category). Insulin prescription was found to be very less (4.5%). Combination of metformin and glimepiride (20.86%) was prescribed most commonly. Most common disease associated with diabetes mellitus was found to be hypertension (35%). Highest prevalence of disease was found to be in the age group of 51 to 60 followed by age group of 41 to 50. Men patients (66.36%) were found to be predominated over women patients (33.64%).

Diabetes is a chronic condition associated with abnormally high levels of sugar (glucose) in the blood. Insulin produced by the pancreas lowers blood glucose. Absence or insufficient production of insulin causes diabetes. Symptoms of diabetes include increased urine output, thirst and hunger as well as fatigue^1^. Diabetes is diagnosed by blood sugar (glucose) testing. The major complications of diabetes are both acute and chronic. Acute complications include dangerously elevated blood sugar, abnormally low blood sugar due to diabetes medications, chronic is related to diseases of the blood vessels (both small and large), which can damage the eye, kidneys, nerves, and heart^2^. Diabetes treatment depends on the type and severity of the diabetes. The two types of diabetes are referred to as type 1 (insulin dependent) and type 2 (non-insulin dependent). Insulin is vital to patients with type 1 diabetes - they cannot live without a source of exogenous insulin. Type 2 diabetes is first treated with weight reduction, a diabetic diet, and exercise when these measures fail to control the elevated blood sugars, oral medications are used. If oral medications are still insufficient, insulin medications are considered^3^. Diabetes is among the leading causes of kidney failure, but its frequency varies between populations and is also related to the severity and duration of the disease^4^. As estimated, 135 million people worldwide have diagnosed diabetes in 1995, and this number is expected to rise to at least 30 million by 2025^5^. Drug utilization studies are powerful exploratory tools to ascertain the role of drugs in society. These studies create a sound sociomedical and health economic basis for healthcare decision making^6^. WHO specifies drug use indicators^7^ for adoption in drug utilization studies. Various guidelines are available that recommended for different classes of drugs to treat diabetes^8^. The present study was conducted to establish the current prescribing pattern of antidiabetic drugs in outpatient pharmacy department, Bombay hospital, Indore (BHI). The BHI, a tertiary critical care hospital, caters the needs of the people from Indore and nearby cities. Drug selection indicators selected for present study includes percentage of male and female patients, number and percentage in various age groups, percentage of one/two drug combination, type of dosage forms, percentage of the utilization of different categories of antidiabetic drugs and percentage of antidiabetic drugs in combination with other category drugs.

The study was performed using prescriptions of around 220 patients in BHI suffering from diabetes. Various age group patients and patients with different types of diabetes have been selected for the study. All the data, which was collected from the out patient department of Pharmacy, were shown in the form of tables. This was a prospective study conducted at a city hospital, Indore, from July to November 2006. Prescriptions of newly registered patients was studied. Patient's data such as the age, name, gender and data on prescribed drugs that include name of drug, dosage form, route of administration, most prescribed drug and so on were recorded on a customized data collection sheet in an approved manner. Each drug was counted only once without considering any change in the regimen.

Out of the 220 prescriptions of antidiabetic drugs studied, 66.36% were for men and 33.64% were for women indicating that men predominated over women (Table 1). Maximum patients with Diabetic Mellitus were of the age group of 51 to 60 years followed by the age group of 41 to 50 years (Table 1). Greater prevalence in this age group may be due to change in life style, lack of exercise and stress. Table 2 indicates that metformin (27%) and glimepiride (22.60%) were the most prescribed drugs. Metformin and glimepiride (20.86%) was prescribed most commonly in combination. Other commonly prescribed drug was pioglitazone (13.91%). Tablet formulation was found as the most commonly prescribed (Table 2) most probably due to the ease of administration. Table 3, shows the description of various category of drugs in different prescriptions. Prescription of oral antidiabetic drugs was found to be maximum (95%).

**TABLE 1:** AGE AND SEX DISTRIBUTION OF DIABETIC PATIENTS

Age groups (Years)	Men	Women	Total
1-20	6	2	8
21-30	12	6	18
31-40	18	14	32
41-50	38	14	52
51-60	40	22	62
61-70	18	12	30
71-80	10	6	16
81-above	4	0	2
Total (%)	66.36	33.64	100

**TABLE 2:** PERCENTAGE OF DRUGS PRESCRIBED

Name of Drug	Dosage Form	Number of times Prescribed	% of total antidiabetic Drugs prescribed
Metformin	Tablet (500mg)	62	27%
Glimepiride	Tablet (1mg, 2mg, 3mg)	52	22.6%
Pioglitazone	Tablet (15mg, 30mg)	32	13.9%
Pioglitazone+Metformin	Tablet (15mg+500mg)	6	2.6%
Glimepiride+Metformin	Tablet (1mg+500mg), (2mg+500mg)	48	20.8%
Pioglitazone+Glimepiride	Tablet (15mg+1mg)	10	4.34%
Miglitol	Tablet (25mg, 50mg)	20	8.69%

**TABLE 3:** DESCRIPTION OF DRUGS PRESENT IN EACH PRESCRIPTION

No of drugs per prescription	No of prescriptions	No of prescriptions containing oral antidiabetic drugs	No of prescription containing insulin	No of prescription containing antihypertensive drugs along with antidiabetics	No of prescription containing other drugs along with antidiabetics
1	36	36	-	-	-
2	76	70	6	24	50
3	62	58	4	24	62
4	28	28	-	18	28
5	18	18	-	12	14
Total	220	210	10	78	77
%	100	95	4.5	35	34.6

Several studies showed that a combination of sulphonylurea with metformin has been most widely used^9^. The present study also showed that a combination of sulphonylurea and metformin was most frequently prescribed (20.86%). Metformin does not promote weight gain and has beneficial effects on several cardiovascular risk factors. Accordingly, metformin is reported to be regarded as the first drug of choice for most patients with Type 2 diabetes^10^. Our study also supported the same conclusion; 50.4% of patients studied received metformin alone and/or in combination with other oral antidiabetic drugs. At present, glibenclamide and glimepiride are the second-generation sulphonylureas most widely used in the United States^11^. In this study among the second-generation sulphonylureas, glimepiride was found to be the most commonly prescribed. Coronary heart disease (CHD) is one of the major causes of death in elderly diabetics^12^. In our study, 35% (Table 3) showed presence of cardiovascular complications other than hypertension and drugs used were nitrates (23.43%) and aspirin (42.18%).

From the data collected, it was observed that among the antidiabetic drug category, drugs were found to be prescribed in following order; metformin>glimepiride>combination of metformin and glimepiride>pioglitazone>miglitol. Among the sulfonylurea category, prescription was found to be maximum for glimepiride followed by glipizide. Among the biguanide category the only drug prescribed was metformin. Among glitazone category the only drug prescribed was pioglitazone.
